# 854 Silver Impregnated Foam Dressings vs Silver Sulfadiazine Dressings in Partial Thickness Burns: Randomized Comparative Study

**DOI:** 10.1093/jbcr/iraf019.385

**Published:** 2025-04-01

**Authors:** Sriranjani Iyer, Smitha Segu, Dr Bhagyashree Hegde

**Affiliations:** Bangalore Medical College; Bangalore Medical College; Bangalore Medical College

## Abstract

**Introduction:**

In order to compare the efficacy and tolerability of silver sulfadiazine (SSD) with an absorbent silver impregnated foam dressing in partial-thickness burns, a randomized control trial was conducted on patients with partial-thickness burn injuries < 40 %, age from 0 to 60.

**Methods:**

Patients were randomized to receive either a Silver impregnated foam dressing (n = 65) applied every 5 to 7 days or an SSD (n = 65) applied every day (Total n=130). There was a 4-week treatment follow up time for both groups. Following 4 follow-ups at the conclusion of every seven days, the healing of wounds and several patient-related factors were evaluated.

**Results:**

Patients had a mean age of 22 years, majority (86.15 %) of which were, less than 30 years of age (n=112). There was faster achievement of end points of healing by the end of 3 weeks with Silver impregnated foam dressing group as compared to the SSD group which showed complete healing by 4 weeks. Mean pain score was significantly lower every week in silver impregnated foam dressing (SSD- 21.5%, Silver impregnated foam- 12.62%).

The mean total number of dressing changes for Silver impregnated foam dressing was 3.04 compared with 22.2 for the SSD group. The ease and pain during application and removal of the dressing, adherence of the dressing, the psychological fear associated with dressing, ease of movement while with the dressing and Stinging or burning sensation, favored Silver impregnated foam dressing.

**Conclusions:**

Silver impregnated foam dressings offers a number of significant benefits compared with SSD.

**Applicability of Research to Practice:**

Silver impregnated foam dressings can revolutionize by healing the wound faster and by reducing the number of dressings and the anxiety associated with dressings as it is well tolerated by patients and encourages undisturbed recovery due to its extended wear period.

**Funding for the Study:**

N/A

